#  

**DOI:** 10.1002/ueg2.12321

**Published:** 2022-10-21

**Late‐breaking Abstracts I**

**Sunday, October 9, 2022 18:00–19:00/A1**

## LB01 | VONOPRAZAN VS. PANTOPRAZOLE IN PREVENTION OF POST ENDOSCOPIC VARICEAL LIGATION ULCER/BLEEDING IN PORTAL HYPERTENSION: A RANDOMIZED CONTROLLED TRIAL

S. A. Lashen ^1^, M. M. Shamseya^2^, A. M. Shamseya^1^, F. H. Hablass^1^

^1^ *Hepatogastroenterology Division, Department of Internal Medicine, Faculty of Medicine, Alexandria University, Alexandria, Egypt* ^2^ *Department of Clinical and Experimental Internal Medicine, Medical Research Institute, Alexandria University, Alexandria, Egypt*

**Contact E‐Mail Address**: sameh.lashen@alexmed.edu.eg

**Introduction:** Up till now, the evidence regarding the use of proton‐pump inhibitors to prevent post‐endoscopic variceal ligation (EVL) ulcers and bleeding is lacking, and the available data are conflicting. Recently, the potassium‐competitor acid blocker vonoprazan has been introduced to the market and has been approved for treatment of acid‐related disorders (erosive esophagitis), and *H. Pylori* infection. Vonoprazan has not been studied as regards its efficacy and safety to prevent post‐EVL ulcer/bleeding.

**Aims and Methods:** The aim was to study the effectiveness and safety of vonoprazan compared to pantoprazole in the prevention of post‐EVL ulcer/bleeding in patients with portal hypertension.

**Material and Methods:** In a randomized, single‐blind, placebo‐controlled design, we included 237 patients with portal hypertension secondary to liver cirrhosis Child‐Pugh class A and B/9 who undergoing EVL. A clinico‐laboratory evaluation was performed at baseline. After having EVL, patients were randomly assigned to receive single daily dose of vonoprazan 20 mg, single daily dose of pantoprazole 40 mg, or to a placebo (no acid suppression therapy). After 14 days, a second look endoscopy was performed to assess the presence of post‐EVL ulcers. Vonoprazan's safety was evaluated during the study.

**Results:** The occurrence of post EVL ulcers was significantly lower among vonoprazan and pantoprazole groups compared to placebo, while there was no difference between vonoprazan and pantoprazole groups. The number of ulcers/case was significantly lower in vonoprazan group compared to pantoprazole and placebo (*p* < 0.001). The ulcer size in vonoprazan and pantoprazole groups was lower compared to placebo (*p* < 0.001). Post‐ligation bleeding occurred among equally among vonoprazan and pantoprazole groups (7.2%, and 7.6% respectively, *p* = 0.92) while bleeding occurred in 18.7% of placebo group (*p* = 0.038). Chest pain and odynophagia incidence was higher in placebo group compared to vonoprazan and pantoprazole groups (*p* < 0.05) (Table 1). The esophageal varices grade and the non‐use of acid suppression therapy were the independent predictors of post EVL ulcer and bleeding in univarmultivariate analysis. There were no patient‐reported adverse reaction related to vonoprazan use. There was no reported laboratory deteriorations in the vonoprazan group.

**Table:** 

	Vonoprazan (*n* = 83)	Pantoprazole (*n* = 79)	Placebo (*n* = 75)	*p*
Number of bands: median (min–max)	5 (3–6)	5 (3–6)	5 (3–6)	0.87
Post EVL ulcers: *n* (%)	24 (28.9)	26 (32.9)	59 (78.7)	<0.001**
Number of ulcers: median (min–max)	2 (1–3)	3 (1–4)^1^	3 (1–4)^1^	<0.001
Max. ulcer length (mm)	9.5 (5–12)	10 (7–12)	14 (8–17)^1^ ^,^ ^2^	<0.001
Max. ulcer width (mm)	5 (3–8)	5.50 (5–8)	9 (6–11)^1^ ^,^ ^2^	<0.001
Chest pain yes: *n* (%)	21 (25.3)	24 (30.4)	46 (61.3)	<0.001
Odynophagia yes: *n* (%)	19 (22.9)	21 (26.6)	34 (45.3)	0.005

*Test used Kruskal Wallis.

**Chi square test, *χ*2 = 47.42.

^1^ Mann‐Whitney *U* test, significant difference from vonoprazan.

^2^ Mann‐Whitney *U* test, significant difference from pantoprazole.

**Conclusion:** A short course of vonoprazan 20 mg single daily dose is as effective and safe as pantoprazole 40 mg/day in the prevention of post‐EVL ulcers and bleeding. Acid suppression is superior to placebo to prevent post‐EVL complications.

**References**

1. Kang SH, Yim HJ, Kim SY, Suh SJ, Hyun JJ, Jung SW, et al. Proton pump inhibitor therapy is associated with reduction of early bleeding risk after prophylactic endoscopic variceal band ligation: a retrospective cohort study. *Medicine (Baltimore)*. 2016;95(8):e2903. 10.1097/MD.0000000000002903. PMID: 26937932; PMCID: PMC4779029.
2. Ashida K, Sakurai Y, Hori T, Kudou K, Nishimura A, Hiramatsu N, et al. Randomized clinical trial: vonoprazan, a novel potassium‐competitive acid blocker, vs. lansoprazole for the healing of erosive oesophagitis. *Aliment Pharmacol Ther*. 2016;43:240–51.
3. Cho E, Jun CH, Cho SB, Park CH, Kim HS, Choi SK, et al. Endoscopic variceal ligation‐induced ulcer bleeding: what are the risk factors and treatment strategies? *Medicine (Baltimore)*. 2017;96(24):e7157. 10.1097/MD.0000000000007157. PMID: 28614248; PMCID: PMC5478333.
4. Lo EA, Wilby KJ, Ensom MH. Use of proton pump inhibitors in the management of gastroesophageal varices: a systematic review. *Ann Pharmacother*. 2015;49(2):207–19. 10.1177/1060028014559244. PMID: 25583938.
5. Kambara H, Hosohata K, Nakatsuji T, Ueno S, Oyama S, Inada A, et al. Safety profile of vonoprazan compared with proton pump inhibitors: insight from a pharmacovigilance study. *Pharmazie*. 2020;75(10):527–30. 10.1691/ph.2020.0604

**Disclosure:** Nothing to disclose.

## LB02 | PROCTOCOLECTOMY WITH PERMANENT ILEOSTOMY IS ASSOCIATED WITH BETTER TRANSPLANT‐FREE SURVIVAL IN PATIENTS WITH PRIMARY SCLEROSING CHOLANGITIS: A RETROSPECTIVE COHORT STUDY

B. Mol ^1^, M. S. van Nieuwamerongen^1^, K. N. van Munster^1^, S. N. van Munster^1^, A. C. de Vries^2^, A. J. P. van der Meer^2^, J. C. Goet^2^, R. K. Weersma^3^, A. Inderson^4^, K. Boonstra^1^, S. van Meer^5^, Y. S. de Boer^1^, B. W. M. Spanier^6^, J. P. H. Drenth^7^, E. S. de Vries^8^, U. Beuers^1^, J. A. Bogaards^9^, C. Y. Ponsioen^1^

^1^ *Gastroenterology and Hepatology*, *Amsterdam UMC*, *Amsterdam*, *The Netherlands*, ^2^ *Gastroenterology and Hepatology, Erasmus MC, Rotterdam, The Netherlands*, ^3^ *Department of Gastroenterology* & *Hepatology, University Medical Center Groningen, Groningen, The Netherlands*, ^4^ *Gastroenterology and Hepatology, Leiden University Medical Center, Leiden, The Netherlands*, ^5^ *Gastroenterology and Hepatology, University Medical Centre Utrecht, Utrecht, The Netherlands*, ^6^ *Rijnstate Hospital Department of Gastroenterology, Arnhem, The Netherlands*, ^7^ *Gastroenterology and Hepatology, Radboudumc University Nijmegen Medical Centre, Nijmegen, The Netherlands*, ^8^ *Isala Hospital, Gastroenterology and Hepatology, Zwolle, The Netherlands*, ^9^ *Epidemiology and Data Science, Amsterdam UMC, Amsterdam, The Netherlands*

**Contact E‐Mail Address**: b.mol@amsterdamumc.nl

**Introduction:** Primary sclerosing cholangitis (PSC) is a severe liver disease of unknown aetiology. Inflammatory bowel disease (IBD), mainly ulcerative colitis (UC), is the strongest known risk factor and seems to be associated with a worse prognosis. After liver transplantation, colectomy—especially proctocolectomy with permanent ileostomy—is associated with decreased rates of recurrent PSC and better graft survival.^1^ At the same time, the effect of colectomy on the disease course of PSC remains unclear.^2^ Available evidence depends either on small patient numbers, may be confounded by unobserved risk factors or colectomy was not considered as time‐depending covariate.

**Aims and Methods:** The aim of our study is to estimate the effect of colectomy on the disease course of PSC by taking time dependency and known risk factors into account.

We conducted a retrospective cohort study based on the Dutch population‐based EpiPSC2 cohort. Patients aged 18 years or older with a diagnosis of PSC were included between 1 January 2008 and 31 December 2021. Transplant‐free survival was defined as liver transplantation or PSC‐related death (excluding colorectal carcinoma). Median survival time was calculated via Kaplan‐Meier estimates. The effect of colectomy was determined as time depending variable via Cox proportional hazards regression and was corrected for the following known risk factors; sex, age at diagnosis, large or small duct PSC, features of auto‐immune hepatitis, time depending IBD status (i.e. no IBD, UC, Crohn's disease [CD] or IBD unspecified [IBD‐U]), and tertiary centre inclusion.

Colectomy was considered in three different manners: (1) any colectomy versus no colectomy; (2) proctocolectomy versus hemi‐/subtotal colectomy versus no colectomy; or (3) proctocolectomy with permanent ileostomy versus any colectomy with a ‘remnant colon’ (i.e. proctocolectomy with pouch, subtotal colectomy or hemicolectomy) versus no colectomy. Selection of colectomy predictor variables was based on the likelihood ratio test. Indication of colectomy (i.e. active disease, dysplasia and colorectal carcinoma or other) was also considered as predictor variable. Events in the first year after colectomy were censored due to a presumed delayed effect of colectomy on disease course.

**Results:** Of the 1341 patients included, 912 (68%) were diagnosed with IBD and 187 (14%) had undergone a colectomy. During a total follow‐up of 14,393 patient years, 370 (28%) patients underwent liver transplantation and 397 (30%) patients died of whom 318 (80%) due to a PSC‐related cause. Median transplant‐free survival time was 20 years. Hazard ratio (HR) was significantly decreased in patients who had undergone proctocolectomy with permanent ileostomy (HR 0.47 [0.24–0.93]) compared to patients without colectomy. This effect was absent in the group with any colectomy with a remnant colon (HR 0.90 [0.67–1.19]). Sensitivity analysis showed no beneficial effect for proctocolectomy with pouch (HR 0.95 [0.62–1.44]). Indication had no apparent effect and was not selected in the final model. Also, presence of UC, CD or IBD‐U was not associated with a worse prognosis compared to absence of IBD (HR 0.99 [0.81–1.21], HR 0.85 [0.64–1.13], HR 0.75 [0.43–1.29] respectively).

**Conclusion:** Our data suggest that proctocolectomy with permanent ileostomy confers protection for liver transplantation and PSC‐related death. This points towards the putative pathophysiologic role of the colonic microbiota in PSC and may open avenues to a better understanding of the aetiology of PSC.

**References**

1. Steenstraten IC, Sebib Korkmaz K, Trivedi PJ, Inderson A, van Hoek B, Rodriguez Girondo MD, et al. Systematic review with meta‐analysis: risk factors for recurrent primary sclerosing cholangitis after liver transplantation. *Aliment Pharmacol Ther*. 2019;49(6):636–43. 10.1111/apt.15148
2. Ong J, Bath MF, Swift C, Al‐Naeeb Y. Does colectomy affect the progression of primary sclerosing cholangitis? A systematic review and meta‐analysis. *Gastroenterol Hepatol Bed Bench*. 2018;11(4):277–83.

**Disclosure:** Nothing to disclose.

## LB03 | LONG‐TERM OUTCOME OF THE POINTER RANDOMIZED TRIAL ON IMMEDIATE VERSUS POSTPONED INTERVENTION FOR INFECTED NECROTIZING PANCREATITIS

C. van Veldhuisen^1,2,3^, N. Sissingh ^3,4^, L. Boxhoorn^5^, S. van Dijk^1^, J. van Grinsven^1^, R. C. Verdonk^6^, M. A. Boermeester^1^, S. A. Bouwense^7^, M. J. Bruno^8^, V. Cappendijk^9^, P. van Duijvendijk^10^, C. van Eijck^11^, P. Fockens^2,5^, H. van Goor^12^, M. Hadithi^13^, J. W. Haveman^14^, M. A. J. M. Jacobs^15^, J. M. Jansen^16^, M. Kop^17^, E. Manusama^18^, S. Mieog^19^, I. Q. Molenaar^20^, V. Nieuwenhuijs^21^, A. C. Poen^22^, J. W. Poley^23^, R. Quispel^24^, T. E. H. Römkens^25^, M. P. Schwartz^26^, T. Seerden^27^, M. Dijkgraaf^28^, M. Stommel^12^, J. W. A. Straathof^29^, N. G. Venneman^30^, R. P. Voermans^2,5^, J. van Hooft^4^, H. C. van Santvoort^20,31^, M. Besselink^1,2^, Dutch Pancreatitis Study Group,

^1^ *Surgery, Amsterdam UMsC, location University of Amsterdam, Amsterdam, The Netherlands*, ^2^ *Amsterdam Gastroenterology Endocrinology Metabolism, Amsterdam, The Netherlands*, ^3^ *Research and Development, St. Antonius Hospital, Nieuwegein, The Netherlands*, ^4^ *Gastroenterology and Hepatology, Leiden University Medical Center, Leiden, The Netherlands,* ^5^ *Gastroenterology and Hepatology, Amsterdam UMC, location University of Amsterdam, Amsterdam, The Netherlands,* ^6^ *Gastroenterology and Hepatology, St. Antonius Hospital, Nieuwegein, The Netherlands,* ^7^ *Surgery, Maastricht UMC+, Maastricht, The Netherlands,* ^8^ *Gastroenterology and Hepatology, Erasmus Medical Center, Rotterdam, The Netherlands,* ^9^ *Radiology, Jeroen Bosch Hospital's Hertogenbosch, The Netherlands*, ^10^ *Surgery, Gelre Hospital, Apeldoorn, The Netherlands,* ^11^ *Surgery, Erasmus Medical Center, Rotterdam, The Netherlands*, ^12^ *Surgery, Radboud University Medical Center, Nijmegen, The Netherlands*, ^13^ *Gastroenterology, Maasstad Hospital, Rotterdam, The Netherlands*, ^14^ *Surgery, University Medical Center Groningen, Groningen, The Netherlands*, ^15^ *Gastroenterology and Hepatology, Amsterdam UMC, location Vrije Universiteit Amsterdam, Amsterdam, The Netherlands*, ^16^ *Gastroenterology and Hepatology, Onze Lieve Vrouwe Gasthuis, Amsterdam, The Netherlands*, ^17^ *Radiology, Amsterdam UMC, location University of Amsterdam, Amsterdam, The Netherlands*, ^18^ *Surgery, Medical Center Leeuwarden, Leeuwarden, The Netherlands*, ^19^ *Surgery, Leiden University Medical Center, Leiden, The Netherlands*, ^20^ *Surgery, University Medical Center Utrecht, Utrecht, The Netherlands*, ^21^ *Surgery, Isala Clinics, Zwolle, The Netherlands*, ^22^ *Gastroenterology and Hepatology, Isala Clinics, Zwolle, The Netherlands*, ^23^ *Gastroenterology and Hepatology, Maastricht UMC+, Maastricht, The Netherlands*, ^24^ *Gastroenterology and Hepatology, Reinier de Graaf Gasthuis, Delft, The Netherlands*, ^25^ *Jeroen Bosch Hospital, Den Bosch, The Netherlands,* ^26^ *Gastroenterology and Hepatology, Meander Medical Center, Amersfoort, The Netherlands*, ^27^ *Gastroenterology and Hepatology, Amphia Hospital, Breda, The Netherlands, ^28^Epidemiology and Data Science, Amsterdam UMC, location University of Amsterdam, Amsterdam, The Netherlands*, ^29^ *Gastroenterology and Hepatology, Máxima Medical Center, Veldhoven, The Netherlands*, ^30^ *Gastroenterology and Hepatology, Medisch Spectrum Twente, Enschede, The Netherlands*, ^31^ *Surgery, St. Antonius Hospital, Nieuwegein, The Netherlands*

**Contact E‐Mail Address**: noorsissingh@hotmail.com

**Introduction:** Patients with infected necrotizing pancreatitis did not benefit from very early catheter drainage in the recent multicenter randomized POINTER trial. Moreover, patients treated with a postponed‐drainage approach required fewer interventions, and over a third of patients were treated with only antibiotics. Whether these beneficial outcomes of postponing drainage persist during long‐term follow‐up is unclear.

**Aims and Methods:** Between August 2015 and October 2019, 104 patients with infected necrotizing pancreatitis were randomly assigned to either immediate catheter drainage or postponed catheter drainage. Here, we re‐evaluated 88 patients who were still alive after the initial 6‐month follow‐up. The primary outcome was a composite of death and major complications. Secondary outcomes included individual components of the primary outcome, pancreas insufficiency, recurrent acute and chronic pancreatitis, interventions (catheter drainage and necrosectomy), hospital stay and quality of life (measured on the 36‐Short Form Health Survey). The analyses were performed according to the intention‐to‐treat principle.

**Results:** During a median follow‐up of 48 months, the primary outcome occurred in 29 (54%) patients in the immediate‐drainage group and 23 (50%) patients in the postponed‐drainage group (relative risk [RR] 1.00, 95% confidence interval [CI] 0.40–2.51). The median number of interventions was four in the immediate‐drainage group and one in the postponed‐drainage group (*p* = 0.001). After the initial 6‐month follow‐up, 16 patients (35%) were still successfully treated without any interventions and only one initially conservatively treated patient received drainage procedures. At the end of follow‐up, quality of life was similar in both groups.

**Conclusion:** Also in long‐term follow‐up, immediate catheter drainage is not superior to postponed drainage in reducing death and major complications in patients with infected necrotizing pancreatitis. Overall, fewer interventions were performed in patients assigned to postponed drainage.

**Reference**

1. Boxhoorn L, van Dijk SM, van Grinsven J, Verdonk RC, Boermeester MA, Bollen TL, et al.; Dutch Pancreatitis Study Group. Immediate versus postponed intervention for infected necrotizing pancreatitis. *N Engl J Med*. 2021;385(15):1372–81. 10.1056/NEJMoa2100826. PMID: 34614330.

**Disclosure:** Nothing to disclose.

## LB04 | A PROCALCITONIN‐BASED ALGORITHM TO GUIDE ANTIBIOTIC USE IN PATIENTS WITH ACUTE PANCREATITIS: THE FINAL RESULTS OF THE PROCAP RANDOMISED CONTROLLED TRIAL

Ajith Siriwardena ^1^, S. Jegatheeswaran^1^, J. Mason^2^, on behalf of the PROCAP Investigators

^1^ *Regional Hepato‐Pancreato‐Biliary Unit, Manchester Royal Infirmary, Manchester, UK*, ^2^ *University of Warwick, Coventry, UK*

**Contact E‐Mail Address**: a.siriwardena@btinternet.com

**Introduction:** Differentiating inflammation from bacterial infection in patients with acute pancreatitis can be difficult. Procalcitonin can distinguish infection from inflammation, and algorithms based on procalcitonin measurement can differentiate bacterial sepsis from a systemic inflammatory response. We aimed to test the hypothesis that a procalcitonin‐based algorithm to guide initiation, continuation, and discontinuation of antibiotics could lead to reduced antibiotic use without an adverse effect on outcome in acute pancreatitis.

**Aims and Methods:** PROCAP was a single‐centre, patient‐blinded, randomised controlled trial done at the Manchester Royal Infirmary (Manchester, UK). Eligible participants were aged 18 years or older and had a clinical diagnosis of acute pancreatitis. Participants were randomly assigned (1:1) to procalcitonin‐guided care or usual care using web‐based randomisation software. The randomisation sequence was stratified by disease severity and admission pathway, using variable block sizes of 4, 6, or 8. Patients, but not clinicians, were masked to group assignment. In the procalcitonin‐guidedcare group, procalcitonin testing was conducted on days 0, 4, 7, and weekly thereafter. Guidance was to stop or not start antibiotics following a test value of <1.0 ng/ml and to start or continue antibiotics following a test value of 1.0 ng/ml or more. In the intervention group, any empirical clinical decision to use antibiotics was preceded by measurement of procalcitonin. Otherwise, both groups received standard care. The primary outcome was use of antibiotics during the index admission to hospital. All analyses were done in the intention‐to‐treat population. This study was registered with the International Standard Randomised Controlled Trial registry, ISRCTN 50584992.

**Results:** Between 29 July 2018, and 13 November 2020, 369 patients were screened, of whom 260 were enrolled and randomly assigned to a treatment group (132 to procalcitonin‐guided care and 128 to usual care). 59 (45%) of patients in the procalcitonin‐guided care group were prescribed antibiotics compared with 79 (63%) in the usual care group (adjusted risk difference −15.6% [95% CI –27.0 to −4.2]; *p* = 0.0071). The odds ratio for the treatment effect was 0·49 (95% CI 0.29–0.83; *p* = 0.0077). There was no significant difference between groups in terms of the number of clinical infections or hospital‐acquired infections per patient. Four (3%) patients in the procalcitonin‐guided care group and three (2%) patients in the usual care group died; all deaths were related to underlying severe pancreatitis. There was no difference in adverse events between the groups.

**Conclusion:** Our findings suggest that procalcitonin‐guided care can reduce antibiotic use without increasing infection or harm in patients with acute pancreatitis. Procalcitonin‐based algorithms to guide antibiotic use should be considered in the care of this group of patients and be incorporated into future guidelines on the management of acute pancreatitis.

**Disclosure:** Ajith Siriwardena has received speaker honoraria from Angiodynamics and BOWA.

## LB05 | PORTAL HYPERTENSION SECONDARY TO CIRRHOSIS IS ASSOCIATED WITH POOR PROGNOSIS, IRRESPECTIVE OF AGE AND AETIOLOGICAL DIFFERENCES‐ A LARGE EPIDEMIOLOGICAL STUDY

S. Kulkarni^1,2^, P. Petryszyn^3^, K. Fiorino^4^, S. Kiddle^5^, E. Nyman^6^, K. Chafekar^7^, S. Chandankhede^7^, S. Agrawal^7^, E. Ekholm^8^, J. Oscarsson^8^, P. Ambery ^8^

^1^ *Late Clinical Development, Cardiovascular, Renal and Metabolism, BioPharmaceuticals R&D, AstraZeneca, Cambridge, UK*, ^2^ *Clinical Pharmacology and Therapeutics, Cambridge University Hospitals, NHS Foundation Trust, Cambridge, UK*, ^3^ *Late Clinical Development, Cardiovascular, Renal and Metabolism, BioPharmaceuticals R&D, AstraZeneca, Warsaw, Poland*, ^4^ *Late Clinical Development, Cardiovascular, Renal and Metabolism, BioPharmaceuticals R&D, AstraZeneca, Gaithersburg, Maryland, USA*, ^5^ *Centre for Artificial Intelligence, Data Science & Artificial Intelligence, R&D, AstraZeneca, Cambridge, UK*, ^6^ *Centre for Artificial Intelligence, Data Science & Artificial Intelligence, R&D, AstraZeneca, Gothenburg, Sweden*, ^7^ *ZS Associates India Pvt. Ltd., Pune, Maharashtra, India*, ^8^ *Late Clinical Development, Cardiovascular, Renal and Metabolism, BioPharmaceuticals R&D, AstraZeneca, Gothenburg, Sweden*

**Contact E‐Mail Address**: Phil.Ambery@astrazeneca.com

**Introduction:** The Global Burden of Disease study 2017 reported over 1.32 million cirrhosis‐related deaths globally, which is approximately 2.4% of all deaths worldwide.^1^ Cirrhosis leads to increased intrahepatic vascular resistance secondary to structural changes along with dynamic vasoconstriction resulting in portal hypertension (pHTN). Clinically significant pHTN, defined as hepatic venous pressure gradient (HVPG) ≥10 mmHg, is associated with life‐threatening complications including ascites, variceal haemorrhage (VH), and hepatic encephalopathy (HE). There is little data published estimating prognosis of pHTN in children compared to adults.^2,3^ The aim of this retrospective cohort study was to assess prevalence of prespecified aetiologies of cirrhosis related pHTN, and further compare the prognosis among children and adults.

**Aims and Methods:** TriNetX's Analytics network, a large federated global electronic healthcare records platform was searched (period of entry of diagnosis: from January 2007 to December 2020) with ICD‐10 codes of prespecified aetiological diagnoses associated with cirrhotic pHTN. Data for two age groups: paediatric ≤18 years (*n* = 1679) and adults >18 years (*n* = 60,579), and event rates for complications including: VH, ascites drain, spontaneous bacterial peritonitis (SBP), hepatorenal syndrome (HRS), routine endoscopy, transjugular intrahepatic portosystemic shunt (TIPS), liver transplant, variceal band ligation (VBL), variceal sclerotherapy and recorded mortality occurring within 24‐month follow‐up period were collected. The adult cohort was subdivided into three subgroups: ‘Most severe’: Patients with cirrhosis and documented VH. ‘Moderate severity’: Patients with cirrhosis and oesophageal varices without VH. ‘Least severe’: Patients with cirrhosis and a diagnosis of pHTN without documented oesophageal varices.

**Results:** Based on the time of documentation of diagnosis, the mean age in the paediatric and adult cohort was 8 and 63 years respectively. The study population was gender balanced in the paediatric cohort (M:F, 52%:48%). In comparison, there was a male preponderance among adults (F: across severity groups 36%–43%), with both cohorts showing dominance of white race (paediatric: 65% and adult: 71%). The most commonly occurring prespecified diagnosis in the paediatric cohort was biliary atresia (23%), followed by primary sclerosing cholangitis (associated in 14%). In the adult cohort, alcoholic liver disease was the most common aetiology (across all severity groups), and was most prevalent in patients who had a VH, followed by chronic viral hepatitis, and non‐alcoholic steatohepatitis. In the paediatric cohort, 33.8% of patients developed VH, 15.9% had VBL and 26.3% underwent liver transplant. Based on a composite of all events of interest (attained in approximately 50% patients), 24‐month survival probability estimate was below 70% in patients developing VH without intervention. In comparison, in the adult cohort, cumulative event rate for VH alone was over 60%. The composite event rate of liver outcomes and death approached 75% and 20% in patients with or without VH respectively, and notably more than half of this incidence was achieved within the first 6 months.

**Conclusion:** In this large real world data, the prognosis of pHTN was poor in both paediatric and adult populations, despite different aetiologies. The very high rates of mortality and morbidity despite currently employed interventions such as beta‐blockers, VBL and TIPs, underscores the unmet need for newer therapies targeting cirrhotic pHTN.

**References**

1. Sepanlou SG, Safiri S, Bisignano C, Ikuta KS, Merat S, Saberifiroozi M, et al. The global, regional, and national burden of cirrhosis by cause in 195 countries and territories, 1990–2017: a systematic analysis for the Global Burden of Disease Study 2017. *Lancet Gastroenterol Hepatol*. 2020;5(3):245–66.
2. Kassam AF, Goddard GR, Johnston ME, Cortez AR, Trout AT, Jenkins TM, et al. Natural course of pediatric portal hypertension. *Hepatol Commun*. 2020;4:1346–52.
3. Asrani SK, Kamath PS. Natural history of cirrhosis. *Curr Gastroenterol Rep*. 2013;15(2):308.

**Disclosure:** AstraZeneca employees have been involved in the inception, design and execution of this epidemiological study. AstraZeneca Employees may own stock/stock options. AstraZeneca are developing newer therapeutic options for cirrhosis related portal hypertension.

**Late‐breaking Abstracts II**

**Monday, October 10, 2022 11:00–12:00/A9**

## LB06 | A NOVEL COMPUTER‐AIDED POLYP DETECTION SYSTEM IN DAILY CLINICAL CARE: AN INTERNATIONAL MULTICENTER, RANDOMIZED, TANDEM TRIAL

M. H. J. Maas ^1^, H. Neumann^2^, H. Shirin^3^, H. Jacob^4^, L. H. Katz^4^, A. Benson^5^, A. Kahloon^6^, E. Soons^1^, R. Hazzan^7^, M. J. Landsman^8^, B. Lebwohl^9^, S. K. Lewis^9^, V. Sivanathan^2^, S. Ngamruengphong^10^, P. D. Siersema^1^

^1^ *Gastroenterology & Hepatology, Radboud University Medical Center, Nijmegen, The Netherlands*, ^2^ *Interventional Endoscopy Center, I. Medizinische Klinik und Poliklinik, University Medical Center Mainz, Mainz, Germany*, ^3^ *Institute of Gastroenterology, Liver, Shamir (Assaf Harofeh) Medical Center, Zerifin, Israel*, ^4^ *The Institute of Gastroenterology, Hadassah Medical Center, Jerusalem, Israel*, ^5^ *Hadassah Medical Center and Faculty of Medicine, Institute of Gastroenterology and Liver Diseases, Hebrew University of Jerusalem, Jerusalem, Israel*, ^6^ *Gastroenterology, Erlanger Health System, Chattanooga, Tennessee, USA*, ^7^ *Haifa Gastroenterology Institute, Assuta Centers, Haifa, Israel*, ^8^ *Gastroenterology, MetroHealth Medical Center, Cleveland, Ohio, USA*, ^9^ *Gastroenterology, Columbia University Irving Medical Center, Cleveland, Ohio, USA*, ^10^ *Department of Gastroenterology, Johns Hopkins University, Baltimore, Maryland, USA*

**Contact E‐Mail Address**: Michiel.maas@radboudumc.nl

**Introduction:** Although colonoscopy is considered the gold standard for detection and removal of premalignant polyps, up to 26% of lesions are missed in tandem studies. Computer‐aided polyp detection (CADe) has shown promise in increasing polyp detection rates.

**Aims & Methods:** The aim of this study was to evaluate a novel CADe system, ‘Magentiq Eye Automatic Polyp Detection System’ (ME‐APDS), for screening and surveillance colonoscopy. A multicenter, randomized, controlled trial (RCT) was conducted at 10 hospitals in Europe, United States and Israel. Patients (18–90 years), referred for screening (non‐FIT) or surveillance colonoscopy, were included. Patients were randomized (1:1) to undergo ME‐APDS‐assisted colonoscopy (MEAC) or conventional colonoscopy (CC). In each arm, a subset of patients was further randomized to undergo tandem colonoscopy; MEAC followed by CC or CC followed by MEAC. Primary objective was adenoma per colonoscopy (APC). Secondary objectives were adenoma detection rate (ADR) and adenoma miss rate (AMR). Outcomes were also evaluated by colonoscopy indication (screening and surveillance), adenoma location, and adenoma size.

**Results:** In total, 950 patients were enrolled, of which 916 completed the assigned colonoscopy, 449 in the MEAC group and 467 in the CC group. APC was higher in MEAC compared to CC (0.70 vs. 0.51, *p* = 0.014; total detected adenomas, 314 vs. 238). Overall, ADR was higher in MEAC compared to CC (37% vs. 30%, *p* = 0.014). ADR was increased in MEAC regardless of colonoscopy indication. Apart from diminutive (0–5 mm) adenomas, MEAC also increased the detection of small (6–9 mm) adenomas compared to CC (14.3% vs. 9.9%, *p* = 0.036). Moreover, an increase in proximal adenoma detection was observed in MEAC compared to CC (46.6% vs. 31.1%, *p* = 0.006). A total of 133 (64 MEAC first, 69 CC first) patients underwent tandem colonoscopy. AMR was 19% in MEAC first compared to 36% in CC first (*p* = 0.024). Use of ME‐APDS had no impact on withdrawal times (*p* = 0.981).

**Conclusion:** ME‐APDS increased adenoma detection (both APC and ADR) in screening and surveillance colonoscopies, and reduced AMR by two‐fold compared to CC. Apart from diminutive lesions, ME‐APDS increased the detection of 6–9 mm adenomas suggesting that this novel CADe system is also able to detect more clinically relevant lesions.

**Disclosure:** Prof. P.D. Siersema: Magentiq Eye LTD. (consulting); Dr. H. Jacob: Magentiq Eye LTD (consulting, options); Dr. Ngamruengphong: Boston Scientific (consulting).

## LB07 | CLIP PLACEMENT DOES NOT PREVENT DELAYED BLEEDING AFTER PROXIMAL COLONIC ENDOSCOPIC MUCOSAL RESECTION (CLIPPER): A MULTICENTER, RANDOMIZED CONTROLLED TRIAL

G. Kemper ^1^, A. Turan^1^, R.‐M. Schreuder^2^, R. Schrauwen^3^, M. Hadithi^4^, P. Didden^5^, B. A. J. Bastiaansen^6^, B. van der Spek^7^, J. Terhaar sive Droste^8^, M. P. Schwartz^9^, W. L. Hazen^10^, J. W. A. Straathof^11^, J. J. Boonstra^12^, A. Alkhalaf^13^, F. J. Voogd^14^, D. Allajar^15^, W. de Graaf^16^, P. Koehestanie^17^, R. Roomer^18^, R. de Ridder^19^, L. M. G. Moons^5^, P. D. Siersema^1^, E. J. M. Van Geenen^1^, Dutch EMR Study Group

^1^ *Gastroenterology and Hepatology, Radboudumc, Nijmegen, The Netherlands*, ^2^ *Gastroenterology, Catharina Hospital, Eindhoven, The Netherlands*, ^3^ *Gastroenterology and Hepatology, Bernhoven, Uden, The Netherlands*, ^4^ *Gastroenterology, Maasstad Hospital, Rotterdam, The Netherlands*, ^5^ *Gastroenterology and Hepatology, University Medical Center Utrecht, Utrecht, The Netherlands*, ^6^ *Gastroenterology and Hepatology, Amsterdam University Medical Centers, location Academic Medical Center, Amsterdam, The Netherlands*, ^7^ *Gastroenterology and Hepatology, Noordwest Hospital Group, Alkmaar, The Netherlands*, ^8^ *Gastroenterology and Hepatology, Jeroen Bosch Hospital's‐Hertogenbosch, The Netherlands*, ^9^ *Gastroenterology and Hepatology, Meander Medical Center, Amersfoort, The Netherlands*, ^10^ *Gastroenterology and Hepatology, Elisabeth TweeSteden Hospital, Tilburg, The Netherlands*, ^11^ *Gastroenterology and Hepatology, Máxima Medisch Centrum, Veldhoven, The Netherlands*, ^12^ *Gastroenterology and Hepatology, Leids University Medical Center, Leiden, The Netherlands*, ^13^ *Gastroenterology and Hepatology, Isala Clinics, Zwolle, The Netherlands*, ^14^ *Gastroenterology and Hepatology, Medical Center Leeuwarden, Leeuwarden, The Netherlands*, ^15^ *Hepatology and Gastroenterology, St. Jansdal Hospital, Hardewijk, The Netherlands*, ^16^ *Gastroenterology, Erasmus Medical Center, Rotterdam, The Netherlands*, ^17^ *Gastroenterology and Hepatology, BRAVIS Hospital, Roosendaal, The Netherlands*, ^18^ *Gastroenterology and Hepatology, Franciscus Gasthuis & Vlietland, Rotterdam, The Netherlands*, ^19^ *Gastroenterology and Hepatology, Maastricht UMC+, Maastricht, The Netherlands*

**Contact E‐Mail Address**: gijs.kemper@radboudumc.nl

**Introduction:** The most common complication after endoscopic mucosal resection (EMR) is delayed bleeding (DB), especially in the proximal colon. Randomized controlled trials in high volume centers suggest that prophylactic clipping (PC) of the resection defect reduces DB in patients with a high DB risk. Guidelines already recommend PC for proximal polyps, despite being technically difficult and expensive.

**Aims and Methods:** We aimed to assess the value of PC in patients receiving EMR for proximal flat polyps in reducing DB in daily clinical practice. This study is a randomized controlled trial conducted in 19 Dutch hospitals with patients referred for EMR of lateral spreading and sessile polyps ≥20 mm in the proximal colon. Patients were randomly assigned (1:1) into groups receiving PC (intervention group) and no PC (control group). PC was standardised in tutorial meetings and entails approximating the resection margins with aligning clips 5–10 mm apart. The primary endpoint is clinically significant DB defined as hematochezia necessitating emergency department presentation, hospitalisation, or re‐intervention within 30 days post‐EMR. This was analysed according to the intention‐to‐treat principle. The trial is registered at ClinicalTrials.gov, NCT03309683.

**Results:** Between 15 May 2018 and 14 December 2021, 356 patients with a median polyp size of 30 mm (IQR 25, 40) were included of whom 179 were randomly assigned to the control group and 177 to the intervention group. DB occurred in 11 (6.1%) patients of the control group and in 16 (8.9%) patients of the intervention group (*p* = 0.30). There were no differences between the control and intervention group in serious adverse events including perforation (two vs. one, *p* = 0.57), post polypectomy syndrome (zero vs. three, *p* = 0.08) and intensive care unit admission (one vs. one). No deaths were reported.

**Conclusion:** PC did not reduce DB in patients undergoing EMR for large lateral spreading and sessile polyps in the proximal colon. This study demonstrates that the burden of laborious and expensive PC is not justified in daily clinical practice.

**Disclosure** The CLIPPER trial is investigator initiated and is financially supported by the Dutch Digestive Foundation (MLDS). Olympus (Japan) contributed Quick Clip Pro endoclips for this trial. P. D. Siersema receives unrestricted grants from Pentax (Japan), Norgine (UK), Motus GI (USA), MicroTech (China), and The eNose Company (Netherlands) and is in the advisory board of Motus GI (USA) and Boston Scientific (USA).

## LB08 | LONG‐TERM COLORECTAL CANCER INCIDENCE AND MORTALITY AFTER SCREENING COLONOSCOPY: THE NORDICC RANDOMIZED TRIAL

M. Bretthauer ^1^, The NordICC Study Group

^1^ *Department of Medicine, Gastrointestinal Endoscopy, University of Oslo, Oslo, Norway*

**Contact E‐Mail Address**: michael.bretthauer@medisin.uio.no

**Introduction:** Colonoscopy is widely used as a colorectal cancer (CRC) screening test. However, precise quantification of screening benefits have been hampered by the absence of randomized trials with long‐term follow‐up. We here report on 10‐year follow‐up results from the NordICC trial.

**Aims and Methods:** The aim of this large‐scale population‐based randomized trial is to assess the effectiveness of colonoscopy screening on CRC incidence and mortality after 10–15 years. Asymptomatic men and women 55–64 years were drawn from the population registries in Poland, Norway, and Sweden between 2009 and 2014 and randomly assigned in a 1:2 ratio to an invitation for screening colonoscopy or standard‐of‐care (no‐screening). We report CRC incidence and mortality after 10‐year in intention‐to‐treat and per‐protocol analyses adjusted for imperfect screening participation.

**Results:** Analyses are based on 28,220 individuals randomized to screening and 56,365 to no‐screening. Screening attendance was 42.0%, 15 patients experienced bleeding after polyp removal. There were no perforations or screening‐related deaths within 30 days. During median 10‐year follow‐up, 259 colorectal cancers were diagnosed in the screening group versus 622 in the no‐screening group. In intention‐to‐treat analyses, 10‐year CRC incidence was 0.98% in the screening group versus 1.20% in the no‐screening group, a risk reduction of 18% (risk ratio 0.82; 95% confidence interval [CI] 0.70–0.93). CRC mortality was 0.28% in the screening group and 0.31% in the no‐screening group (RR 0.90; 95% CI 0.64–1.16). The number needed to invite for screening to prevent one CRC was 455 (95% CI 270–1429). In adjusted per‐protocol analyses, CRC incidence was reduced from 1.22% to 0.84% (RR 0.69; 95% CI 0.55–0.83) and CRC mortality from 0.3% to 0.15%. (RR 0.50; 95% CI 0.27–0.77).

**Conclusion:** Once‐only colorectal cancer screening with colonoscopy reduces colorectal cancer incidence over 10 years.

**Disclosure** Nothing to disclose.

## LB09 | ROLE OF ARTIFICIAL INTELLIGENCE IN COLONOSCOPY DETECTION OF ADVANCED LESIONS

C. Mangas‐Sanjuan ^1^, L. de‐Castro^2^, J. Cubiella^3^, P. Diez Redondo^4^, A. Suárez González^5^, M. Pellisé Urquiza^6^, N. Fernández Fernández^7^, S. Zarraquiños Martínez^8^, H. Núñez Rodríguez^9^, V. Alvarez Garcia^5^, O. Ortiz^10^, N. Sala Miquel^11^, P. Zapater^12^, R. Jover^11^, CADILLAC Study Investigators

^1^ *Gastroenterology, Hospital General Universitario de Alicante, Alicante, Spain*, ^2^ *Hospital Álvaro Cunqueiro, Vigo, Spain*, ^3^ *Department of Gastroenterology, Complejo Hospitalario de Ourense, Ourense, Spain*, ^4^ *Gastroenterology, Universitary Hospital Rio Hortega, Vallodolid, Spain*, ^5^ *Hospital Central de Asturias, Oviedo, Spain*, ^6^ *Gastroenterology, Hospital Clinic, Barcelona, Spain*, ^7^ *Gastroenterology, Hospital Alvaro Cunqueiro, Vigo, Spain*, ^8^ *Gastroenterology, Complexo Hospitalario Universitario de Ourense, Ourense, Spain*, ^9^ *Gastroenterology, Hospital Universitario Rio Hortega, Valladolid, Spain*, ^10^ *Gastroenterology, Hospital Clinic Barcelona, Barcelona, Spain*, ^11^ *Hospital General Universitario Dr. Balmis, Alicante, Spain*, ^12^ *Hospital General Universitario de Alicante, Alicante, Spain*

**Contact E‐Mail Address**: cmangassanjuan@gmail.com

**Introduction:** Electronic computer‐aided detection (CADe) systems have demonstrated its role in improving adenoma detection rate at the expense of detecting small polyps and non‐advanced adenomas, whereas its ability to improve detection of advanced and more significant lesions is doubtful. Fecal immunochemical test‐based colorectal (CRC) screening is able to select people with a higher risk of advanced lesions, and, therefore, it is the ideal setting to demonstrate the potential ability of CADe to increase detection of advanced lesions. Moreover, none of the studies published until now have been powered to detect differences in the detection of these advanced lesions.

**Aims and Methods:** This is a multicenter, parallel, controlled, randomized trial, where consecutive individuals presenting for colonoscopy after a positive FIT for the first time within the Spanish population‐based CRC screening program were initially enrolled. Individuals with complete colonoscopy and adequate colon cleansing were finally included and randomly assigned to receive colonoscopy with or without assistance of the CADe system. Patients were excluded in case of personal history of CRC, inflammatory bowel disease, colorectal surgery, terminal illness or severe disease, familial CRC or family history of inherited CRC syndrome, and lack of informed written consent.

Primary outcome was detection of advanced colonic lesion (advanced adenoma and/or advanced serrated polyp). Secondary outcomes included detection of adenomas, serrated polyps, right‐sided and non‐polypoid lesions. The CADe device used is a dedicated convolutional neural network system (GI‐Genius, Medtronic) for polyp detection.

**Results:** A total of 3399 individuals were eligible for enrolment between April 2021 and March 2022. Among them, 186 patients were excluded during colonoscopy according to the predefined exclusion criteria. 3213 patients (1610 in the CADe group and 1603 in the control group) were finally included for analysis. Mean age was 61 (SD, 6) years, 53.4% (*n* = 1717) of individuals were men, and mean BBPS score was 7.8 (SD, 1.3), without differences between both groups.

There were no differences in the primary outcome of the study, detection of advanced colonic lesions (CADe 34.8% vs. control group 34.6%; aOR 1.01; 95% CI: 0.87–1.16) also the mean number of advanced colonic lesions per colonoscopy was not increased using CADe (CADe 0.54 ± 0.95 vs. control group 0.52 ± 0.95; β‐coefficient 0.02; 95% CI −0.05 to 0.09).

CADe significantly increased visualisation of adenomas proximal to splenic flexure (43.7% vs. 38.2%; aOR 1.26; 95% CI 1.09–1.46) and non‐polypoid lesions (27.8% vs. 24.1%, aOR 1.21; 95% CI 1.03–1.42). Regarding serrated lesions, CADe helped to increase the proportion of procedures with a lesion ≤5 mm (15.3% vs. 12.4%; aOR 1.28; 95% CI 1.04–1.57) and also to detect large (≥10 mm) serrated polyps (5.3% vs. 3.8%; aOR 1.44, 95% CI 1.03–2.03).

**Conclusion:** We found that colonoscopy assisted by CADe is not able to improve the detection of advanced colonic lesions. However, there is an increase in the detection of small adenomas, right‐sided or non‐polypoid lesions. Moreover, the use of CADe significantly improves detection of small and large serrated polyps. Evaluation of the performance of artificial intelligence applications is dynamic, and our results show the need of improvement of future versions of this technology with the use of larger and more variate datasets for training of deep learning systems and subsequent evaluations of these new devices in clinical practice, using large and adequately powered randomized control trials.

**Disclosure:** Nothing to disclose.

## LB10 | EFFECT OF REAL‐TIME COMPUTER‐AIDED POLYP DETECTION SYSTEM (ENDOAID) ON ADENOMA DETECTION IN ENDOSCOPIST‐IN‐TRAINING: A SINGLE‐BLIND RANDOMIZED CONTROLLED TRIAL (ENDOAID‐TRAIN STUDY)

H. S. L. Lau ^1,2^, C. L. J. Ho^1^, C. T. J. Lai^1^, A. H. Y. Ho^1^, C. W. K. Wu^1^, V. W. H. Lo^1^, C. M. S. Lai^3^, M. W. Scheppach^2,4^, F. Sia^2^, K. H. K. Ho^2^, X. Xiao^2^, T. Y. T. Lam^5^, H. Y. H. Kwok^1^, H. C. H. Chan^1^, R. N. Lui^1^, T. T. Chan^1^, M. T. L. Wong^1^, M. F. Ho^3^, R. C. W. Ko^3^, S. Chu^3^, K. Futaba^3^, S. S. M. Ng^2,3^, H. C. Yip^2,3^, R. S. Y. Tang^1,2^, V. W. S. Wong^1,2^, J. Y. W. Lau^2,3^, F. K. L. Chan^1,2^, P. W. Y. Chiu^2,3^, ENDOAID‐TRAIN Study Group

^1^ *Department of Medicine and Therapeutics, The Chinese University of Hong Kong, Hong Kong, Hong Kong, China (SAR)*, ^2^ *Institute of Digestive Disease, The Chinese University of Hong Kong, Hong Kong, Hong Kong, China (SAR)*, ^3^ *Department of Surgery, The Chinese University of Hong Kong, Hong Kong, Hong Kong, China (SAR)*, ^4^ *Gastroenterology, Faculty of Medicine, University of Augsburg – Germany, Augsburg, Germany*, ^5^ *Stanley Ho Big Data Decision Analytics Research Centre, The Chinese University of Hong Kong, Hong Kong, Hong Kong, China (SAR)*

**Contact E‐Mail Address**: louis321hk@gmail.com

**Introduction:** Insufficient endoscopist experience is associated with a lower adenoma detection rate (ADR). The effect of real‐time computer‐aided polyp detection systems (CADe) on inexperienced endoscopists‐in‐training during colonoscopies remains unknown.

**Aims and Methods:** We performed a single‐blind, parallel‐group, randomized controlled trial in Hong Kong from April 2021 to July 2022. Consecutive eligible subjects undergoing colonoscopies for screening/surveillance/diagnostic purposes were randomized in 1:1 ratio to receive colonoscopies with the use of real‐time CADe system (ENDOAID, Olympus) or without it (control) during the withdrawal phases. All colonoscopies were performed by endoscopists‐in‐training with a personal experience of <500 procedures and less than 3 years. Randomisation was stratified by age, gender and endoscopist experience (beginner vs. intermediate level, <200 vs. 200–500 procedures). Image enhancement or device assistance was not allowed. Subjects with incomplete colonoscopies or low Boston Bowel Preparation Scale (BBPS) were excluded. Treatment allocation was blinded to outcome assessors (pathologists and data analysts) but not endoscopists. The primary outcome was ADR and secondary outcomes included ADR of different sizes and sites, mean number of adenomas, advanced ADR, sessile serrated lesion detection rate, polyp detection rate and non‐neoplastic resection rate. All outcomes were defined by the histopathology of specimens according to the Vienna classification.

**Results:** Among 766 subjects included, 386 and 380 subjects were randomized to CADe and control groups respectively. Baseline characteristics were comparable. The overall ADR was significantly higher in CADe group than control group (57.0% vs. 43.9%, *p* < 0.001). (Table 1) The ADRs for <5 mm (40.2% vs. 24.5%, *p* < 0.001) and 5–10 mm adenomas (36.5% vs. 28.7%, *p* = 0.025) were significantly higher in CADe group. The ADRs were significantly higher in CADe group at both right‐sided (41.7% vs. 30.3%, *p* = 0.001) and left‐sided colon (33.9% vs. 27.1%, *p* = 0.048). The ADRs were significantly higher among beginner (57.9% vs. 39.3%, *p* = 0.008) and intermediate level endoscopists (56.6% vs. 45.8%, *p* = 0.015) in CADe group. Mean number of adenomas per colonoscopy was significantly higher in CADe group (1.46 vs. 0.85, *p* < 0.001). Non‐neoplastic resection rate was higher in CADe group (51.8% vs. 34.5%, *p* < 0.001). There was no significant difference for advanced ADR (7.3% vs. 8.4%) and sessile serrated lesion detection rate (2.1% vs. 1.8%). By multivariable analysis, the use of CADe was an independent factor for a higher ADR (odds ratio 1.56, 95% CI 1.13–2.18, *p* = 0.008), while other significant factors included advanced age, male gender, total withdrawal time and endoscopist experience.

**TABLE 1:** Baseline characteristics and outcomes between computer‐aided polyp detection systems (CADe) and control groups

	CADe group (*n* = 386)	Control group (*n* = 380)	
Demographics			
Age (mean, SD)	66.0 (10.1)	65.4 (11.3)	
Gender, male (*n*, %)	205 (53.1)	211 (55.5)	
Ethnicity, Chinese (*n*, %)	384 (99.5)	376 (98.9)	
Smoker, current (*n*, %)	42 (10.9)	38 (10.0)	
Drinker, current (*n*, %)	37 (9.6)	32 (8.4)	
Family history of colorectal cancer (*n*, %)	72 (18.7)	55 (14.5)	
Colonoscopy details			
Indications (*n*, %)			
Screening	28 (7.3)	23 (6.1)	
Surveillance	126 (32.6)	121 (31.8)	
Symptomatic/diagnostic	232 (60.1)	236 (62.1)	
Endoscope models (*n*, %)			
HQ‐290 series	376 (97.4)	374 (98.4)	
CV‐1500/1200 series	10 (2.6)	6 (1.6)	
Endoscopist specialty *(n*, %)			
Gastroenterologists	304 (78.8)	290 (76.3)	
Surgeons	82 (21.2)	90 (23.7)	
Endoscopist experience (*n*, %)			
Beginners (<200 procedures)	114 (29.5)	107 (28.2)	
Intermediate (200–500 procedures)	272 (70.5)	273 (71.8)	
Boston bowel preparation scale (mean, SD)			
Right	2.43 (0.50)	2.45 (0.50)	
Transverse	2.69 (0.46)	2.69 (0.46)	
Left	2.72 (0.45)	2.70 (0.46)	
Caecal intubation time, minutes (mean, SD)	9.84 (7.71)	9.82 (8.17)	
Total withdrawal time, minutes (mean, SD)	18.78 (12.06)	16.43 (11.45)	
Outcomes			*p* value*
Overall ADR (*n*, %)	220 (57.0)	167 (43.9)	<0.001
ADR by size (*n*, %)			
<5 mm	155 (40.2)	93 (24.5)	<0.001
5–10 mm	141 (36.5)	109 (28.7)	0.025
>10 mm	7 (1.8)	14 (3.7)	0.173
ADR by location (*n*, %)			
Right colon (from caecum to transverse colon)	161 (41.7)	115 (30.3)	0.001
Left colon (from splenic flexure to rectum)	131 (33.9)	103 (27.1)	0.048
ADR by experience (*n*, %)			
Beginners (*n* = 221)	66 (57.9)	42 (39.3)	0.008
Intermediate (*n* = 545)	154 (56.6)	125 (45.8)	0.015
Mean number of adenomas per colonoscopy (SD)	1.46 (2.05)	0.85 (1.53)	<0.001
<5 mm	0.76 (1.34)	0.37 (0.83)	<0.001
5–10 mm	0.68 (1.31)	0.44 (0.94)	0.010
>10 mm	0.02 (0.13)	0.04 (0.21)	0.112
Right colon	0.89 (1.44)	0.48 (1.05)	<0.001
Left colon	0.57 (1.11)	0.37 (0.78)	0.015
Advanced ADR (*n*, %)	28 (7.3)	32 (8.4)	0.641
Sessile serrated lesion detection rate (*n*, %)	8 (2.1)	7 (1.8)	0.999
Polyp detection rate (*n*, %)	292 (75.6)	232 (61.1)	<0.001
Non‐neoplastic resection rate (*n*, %)	200 (51.8)	131 (34.5)	<0.001

Abbreviations: ADR, adenoma detection rate; SD, standard deviation.

*p‐values were calculated by Pearson Chi‐square test or Wilcoxon Rank‐Sum test where appropriate.

**Conclusion:** The use of real‐time CADe during colonoscopies in endoscopists‐in‐training could increase the overall ADR, especially for small‐to‐medium size adenomas, in different levels of experience (ClinicalTrials.gov number: NCT04838951).

**Disclosure:** This study received research grant support from Asian Endoscopy Research Forum (AERF) and Asian Novel Bio‐Imaging and Intervention Group (ANBIIG).

**Late‐breaking Abstracts III**

**Tuesday, October 11, 2022 11:00–12:00/B9**

## LB11 | TO THE SOURCES OF INFLAMMATORY BOWEL DISEASE: PRE‐HISTORIC INTRODUCTION OF IL‐23 RECEPTOR RELATED GENETIC SUSCEPTIBILITY TO INFLAMMATION INTO TODAY'S EUROPEAN POPULATION

B. Krause‐Kyora^1^, G. G. Torres^2^, N. da Silva^1^, A. Nebel^1^, S. Schreiber ^1,3^, Archaeological Civilization Disease Consortium (ACDC)

^1^ *Institute for Clinical Molecular Biology, Kiel University, Kiel, Germany*, ^2^ *Institute Clinical Molecular Biology, Kiel University, Kiel, Germany,* ^3^ *Department of Internal Medicine I, University Hospital Schleswig‐Holstein, Kiel, Germany*

**Contact E‐Mail Address**: s.schreiber@mucosa.de

**Introduction:** Inflammatory bowel disease (IBD) is considered a novel human disorder with manifestation triggered in genetically susceptible individuals by lifestyle of late Western industry civilisations. Genetic variants in the IL‐23 receptor convey a particularly high risk among the >250 known genetic variants and loci. Analysis of ancient European populations that inhabited Europe at low density >15,000 years ago and that underwent massive selection by environment and substantial population admixtures may give important clues how today's risk variants were introduced into the Caucasian genome.

**Aims and Methods:** Sequencing data were obtained from a collection of 2445 ancient individuals from Western Eurasia, Near East and Russian Steppe. In 172 ancient individuals from the early (5500‐4500 BC) and late Neolithic (3500‐2900), that were grouped into nine samples by archaeological context, dating and ancestry, additional whole genome shotgun and capture data were generated for nine SNPs in IL‐23R (rs11209026, rs11209003, rs2902440, rs7517847, rs11465804, rs11581607, rs80174646, rs1343151, rs6669582). For precise allele frequencies, we used diploid instead of pseudo‐haploid genotyping. Sequencing alignment:Bcftools v1.12 (Li, 2011) producing diploid genotypes on X positions in the IL23R locus. QC‐Filtering criteria: MQ > 20; BAQ > 20; QUAL ≥20; GQ > 20; DP > 3, remooval of Indels and high density markers, SNV pruning:plink v1.9. Deviations from HWE (*p* < 0.0001) were removed. Admixture‐informed analysis of allele frequency trajectories was followed by a selection scan using Tajima D analysis (15 kb windows across chromosome 1 in select high coverage samples in comparison with the Tajima D value of a 30 kb region around rs11209026), and FST statistics.

**Results:** The IBD protective IL‐23R allele rs11209026‐A, which is strongly associated with IBD and is also anti‐inflammatory in several model systems, was completely absent in the European Hunter‐Gatherers, the European population >10–15,000 years ago. It was first detected (population frequency: 21%) in Neolithic Anatolian Farmers (that about 13,000 BC split from Hunter‐Gatherers by adopting farming lifestyle) and was then found in population frequencies of 10%–16% in Early Central European Farmers. Genomes of the Steppe population (horseback based nomadic life‐style), that overwhelmed the European Farmers in the Chalcolitic and early Bronze‐Age showed absence of the variant. With the massive invasion of the Steppe population into continental Europe shaping today's European ancestry at about 2600 BC, frequency of rs11209026‐A continuously decreased from the neolithic period until it reached the present level of about 6%, which is steady since the Middle Ages. Today's population frequency of rs11209026‐A shows a North‐South and East‐West gradient that reflects penetration of the Steppe population into Europe but also parallels today's gradient in the incidence of IBD.

**Conclusion:** The advent of IL‐23R variants may result from selection pressure due to farming‐lifestyle. Lifestyle of early farmers with nutrition changes (carbohydrates), zoonotic infections and the proximity to animal microbiomes by co‐housing with lifestock may have resulted in an advantage of genetically anchored anti‐inflammation (i.e. protective IL23R variants) during a period with intense selective pressures by nutrition, famine and infections. Strong populations admixture in the early bronze‐age from individuals from the Steppe that were not underlying this selection and were free of anti‐inflammatory IL‐23R variants has resulted in today's genetic susceptibility pattern across Europe.

**Disclosure:** Funding by the DFG Excellence cluster ‘Precision Medicine in Inflammation’.

## LB12 | POLIPROTECT VS OMEPRAZOLE IN THE RELIEF OF HEARTBURN, EPIGASTRIC PAIN AND BURNING IN NERD AND EPS PATIENTS: A RANDOMIZED, DOUBLE‐BLIND, DOUBLE DUMMY, REFERENCE PRODUCT CONTROLLED, PARALLEL GROUP, NON‐INFERIORITY CLINICAL STUDY

Enrico S. Corazziari ^1^, A. Gasbarrini^2^, L. D'Alba^3^, V. D'Ovidio^4^, O. Riggio^5^, S. Passaretti^6^, Bruno Annibale^7^, M. Cicala^8^, A. Repici^9^, G. Bassotti^10^, C. Ciacci^11^, A. Di Sabatino^12^, M. Neri^13^, M. C. Bragazzi^14^, E. Ribichini^15^, G. Radocchia^16^, M. Marazzato^16^, S. Schippa^16^, D. Badiali^17^

^1^ *IRCCS Humanitas Research Hospital, Rozzano, Dept. of Gastroenterology, Rozzano, Italy*, ^2^ *Internal Medicine, Gastroenterology and Liver Diseases, Fondazione Policlinico Universitario Gemelli IRCCS, Università’ Cattolica, Rome, Italy*, ^3^ *Department of Gastroenterology and Digestive Endoscopy, Azienda Ospedaliera San Giovanni Addolorata, Rome, Italy*, ^4^ *Unit of Gastroenterology and Digestive Endoscopy, S.Eugenio Hospital, Rome, Italy*, ^5^ *Dipartimento di Medicina Traslazionale e di Precisione, Sapienza University of Rome, Rome, Italy*, ^6^ *Gastroenterology, San Raffaele University Hospital, Milano, Italy*, ^7^ *University Hospital S. Andrea, Rome, Medical and Surgical sciences, Rome, Italy*, ^8^ *Gastroenterologia, Università Campus Bio Medico, Roma, Italy*, ^9^ *Dept. of Gastroenterology, Ist. Clinico Humanitas Rozzano, Milano, Italy*, ^10^ *Gastroenterology & Hepatology Section, Department of Medicine & Surgery, University of Perugia, Perugia, Italy*, ^11^ *Medicine Surgery Dentistry, Scuola Medica Salernitana, University of Salerno, Salerno, Italy*, ^12^ *Prima Clinica Medica, IRCCS Policlinico S. Matteo, Pavia, Italy*, ^13^ *Department of Medicine and Ageing Sciences and Center for Advanced Studies and Technology (CAST), “G. d'Annunzio” University of Chieti‐Pescara, Chieti, Italy*, ^14^ *Sapienza University of Rome, Dip. Di scienze e Biotecnologia Medico Chirurgiche, Rome, Italy*, ^15^ *Gastroenterology and Gastrointestinal Endoscopy Unit, IRCCS San Raffaele Scientific Institute, Vita‐Salute San Raffaele University, Milan, Italy*, ^16^ *Department of Public Health and Infectious Diseases, Sapienza University of Rome, Rome, Italy*, ^17^ *Department of Translational and Precision Medicine, Sapienza University of Rome, Rome, Italy*

**Contact E‐Mail Address**: enrico.corazziari@uniroma1.it

**Introduction:** Mucosal protective agents (MPA) adhere to the esophagogastric epithelium, and, reinforcing its permeable barrier, protect it from acid and other offensive agents. Non‐erosive reflux disease (NERD) and epigastric pain syndrome (EPS) patients use MPA alone or as add‐on treatment to increase the often inadequate response of the standard reference therapy with proton‐pump inhibitors,^1^ which are not devoid of adverse events (AEs) and account for significant cost expenditure. No controlled studies have so far assessed the benefit of MPA as the only treatment of heartburn and epigastric pain and burning in NERD and EPS patients

**Aims and Methods:** This randomized, controlled, double‐blind, double‐dummy, non‐inferiority trial, evaluated the efficacy and safety of a CE marked class IIa medical device MPA Poliprotect, (made of a polysaccharide fraction from *Aloe vera*, *Malva sylvestris* and *Althaea Officinalis*, the natural minerals Limestone and Nahcolite, and a flavonoid fraction from *Glycyrrhiza glabra*, *Matricaria recutita*: Aboca, Sansepolcro, Italy), versus Omeprazole (Doc Generici Srl, Milano, Italy) in the relief of heartburn and epigastric pain/burning in NERD (Montreal criteria) and/or EPS (Rome III criteria)^2‐4^ patients with a negative upper endoscopy performed in the last 3 years.

After a 2‐week screening/wash‐out period patients were randomized at V0 into either Omeprazole group (Omeprazole verum, 20 mg q.d 30’ before breakfast + Poliprotect placebo) or Poliprotect group (Omeprazole placebo + Poliprotect verum), for a double‐blind 4‐week period (V0–V2). In the first 2 weeks (V0–V1), one 1.55 g Poliprotect tablet was taken five times a day (30’ after breakfast, lunch, and dinner; at midafternoon; before going to bed), whereas, in the following 2 weeks, Poliprotect intake was on‐demand. Magaldrate oral gel (80 mg/ml, Takeda Italia S.p.A.) was allowed as rescue medication. Daily diaries recording symptom VAS score, concomitant medications, investigational products, and rescue therapy intake, were filled by each patient. The gut microbiome was assessed at V0 and V2.

The primary and secondary efficacy endpoints were the comparison between the two groups of heartburn and/or epigastric pain/burning severity from baseline to V1 and V2, respectively, as measured by means of a 100 mm VAS. A non‐inferiority margin of −11 was considered clinically acceptable and determined accordingly to technical recommendation.^5^

**Results:** Patients (*N* = 275, F 62%, Mean age 49 years) randomized to Omeprazole and Poliprotect arm did not differ at baseline for demographic and other relevant characteristics. Compliance was equal or greater than 90% in both treatment groups. Two‐week treatment with Poliprotect proved non‐inferior to omeprazole 20 mg q.d. in symptom relief (between‐group difference of symptom score change: −5.4, 95% CI −9.9 to −0.1; −6.2, 95% CI −10.8 to −1.6; ITT and PP populations, respectively).

Poliprotect benefit continued unaltered after shifting to on‐demand intake, without gut microbiota variation. The initial omeprazole benefit was maintained against a significantly higher number of rescue medicine sachets used (mean, 95% CI: Poliprotect 3.9, 2.8–5.0; Omeprazole 8.2, 4.8–11.6) and was associated to oro‐mucosal genera outgrowth in the gut. No relevant AEs were reported in both treatment arms.

**Conclusion:** Poliprotect proved non‐inferior to the standard dose of Omeprazole in promptly relieving heartburn and epigastric pain/burning in NERD and EPS patients, without affecting gut microbiota.

(ClinicalTrials.gov: NCT03238534. EudraCT Database: 2015‐005216‐15)

**References**

1. Savarino V, Pace F, Scarpignato C. Randomised clinical trial: mucosal protection combined with acid suppression in the treatment of non‐erosive reflux disease ‐ efficacy of Esoxx, a hyaluronic acid‐chondroitin sulphate based bioadhesive formulation. *Aliment Pharmacol Ther*. 2017;45(5):631–42. (In Eng). 10.1111/apt.13914
2. Vakil N, van Zanten SV, Kahrilas P, Dent J, Jones R. The Montreal definition and classification of gastroesophageal reflux disease: a global evidence‐based consensus. *Am J Gastroenterol*. 2006;101(8):1900‐20; quiz 1943. (In Eng). 10.1111/j.1572–0241.2006.00630.x
3. Modlin IM, Hunt RH, Malfertheiner P, Moayyedi P, Quigley EM, Tytgat GN, et al. Diagnosis and management of non‐erosive reflux disease—the Vevey NERD Consensus Group. *Digestion*. 2009;80(2):74–88. (In eng). 10.1159/000219365
4. Tack J, Talley NJ, Camilleri M, Holtmann G, Hu P, Malagelada JR, et al. Functional gastroduodenal disorders. In: Drossman DA, Corazziari E, Delvaux M, Spiller RC, Talley NJ, Thompson WG, et al., editors. Rome III. The functional gastrointestinal disorders. McLean: Degnon Associates Inc; 2006. p. 419–86.
5. D'Agostino RB Sr, Massaro JM, Sullivan LM. Non‐inferiority trials: design concepts and issues ‐ the encounters of academic consultants in statistics. *Stat Med*. 2003;22(2):169–86. (In Eng). 10.1002/sim.1425

**Disclosure** Enrico Stefano Corazziari and Bruno Annibale are consultants to Aboca Società Agricola S.p.A.

## LB13 | FOUR SARS‐COV‐2 VACCINE DOSES OR HYBRID IMMUNITY IN PATIENTS WITH IMMUNE‐MEDIATED INFLAMMATORY DISEASES ON IMMUNOSUPPRESSIVE THERAPIES. A PROSPECTIVE, OBSERVATIONAL COHORT STUDY

K. H. Bjørlykke^1,2^, H. S. Ørbo^2,3^, A. T. Tveter^3^, T. T. Tran^2,4^, J. Sexton^3^, I. Jyssum^2,3^, I. E. Christensen^2,3^, G. B. Kro^5^, T. K. Kvien^2,3^, J. Jahnsen^1,2^, L. A. Munthe^2,4,6^, A. Chopra^4^, D. J. Warren^7^, G. Grødeland^2,4^, E. A. Haavardsholm^2,3^, S. Mjaaland^8^, S. A. Provan^3^, J. T. Vaage^2,4^, S. W. Syversen^3^, G. Lovik Goll^3^, K. K. Jørgensen ^1^

^1^ *Department of Gastroenterology, Akershus University Hospital, Lørenskog, Norway*, ^2^ *Institute of Clinical Medicine, University of Oslo, Oslo, Norway*, ^3^ *Center for treatment of Rheumatic and Musculoskeletal Diseases (REMEDY), Diakonhjemmet Hospital, Oslo, Norway*, ^4^ *Department of Immunology, Oslo University Hospital, Oslo, Norway*, ^5^ *Department of Microbiology, Oslo University Hospital, Oslo, Norway*, ^6^ *KG Jebsen Centre for B Cell Malignancies, University of Oslo, Oslo, Norway*, ^7^ *Department of Medical Biochemistry, Oslo University Hospital, Oslo, Norway*, ^8^ *Norwegian Institute of Public Health, Oslo, Norway*

**Contact E‐Mail Address**: Kristin.Kaasen.Jorgensen@ahus.no

**Introduction:** Data on response and safety of repeated SARS‐CoV‐2 vaccinations and hybrid immunity is needed in patients with inflammatory bowel disease and other immune‐mediated inflammatory diseases (IMIDs) on immunosuppressive therapy to further develop vaccination strategies in this vulnerable population.

**Aims and Methods:** The aims of the study were to evaluate humoral immune response and safety of four SARS‐CoV‐2 vaccine doses and hybrid immunisation in IMID patients on immunosuppressive therapy as compared to healthy controls, and to compare humoral immune responses induced by fourth (booster) dose BNT162b2 (full‐dose) versus mRNA‐1273 (half‐dose) vaccine. This prospective observational Norwegian study of vaccine response to COVID‐19 (Nor‐vaC)^1^ included adult IMID patients on immunosuppressive therapy, receiving four SARS‐CoV‐2 vaccine doses (vaccine group) or three vaccine doses followed by SARS‐CoV‐2 Omicron infection (hybrid group), and healthy controls receiving three vaccine doses (control group). Antibodies to the receptor‐binding domain of SARS‐CoV‐2 spike proteins were assessed 2–4 weeks following vaccination or Omicron infection.

**Results:** The study included 656 patients (130 Crohn's disease [CD], 95 ulcerative colitis, 209 rheumatoid arthritis, 107 spondyloarthritis, and 115 psoriatic arthritis, median age 55 years [IQR 44–65]; 55% women). In the vaccine group (*n* = 492), anti‐Spike antibody levels were higher following the fourth vaccine dose than the third dose (median 6846 BAU/ml [IQR 3593–12,073] vs. 6016 BAU/ml [1857–9453], *p* < 0.001), but lower than in the control group (*n* = 149) vaccinated with three doses (median 7595 BAU/ml [IQR 5915–12,001], *p* = 0.002). There was no significant difference in anti‐Spike antibody levels between fourth (booster) dose vaccination with BNT162b2 versus mRNA‐1273 (median 6687 BAU/ml [IQR 3582–12,292] vs. 7031 BAU/ml [3643–11,755], *p* = 0.98).

In the hybrid group (*n* = 164), anti‐Spike antibody levels were 3.5 times higher compared to the vaccine group (median 23,991 BAU/ml [IQR 11,449–35,564] vs. 6846 BAU/ml [3593–12,073], *p* < 0.001), and three times higher compared to the control group (7595 BAU/ml [IQR 5915–12,001], *p* < 0.001).

In the vaccine group, the finding of higher anti‐Spike antibody levels following the fourth versus the third dose was consistent across diseases. Tumour necrosis factor inhibitor therapy, both as monotherapy and combination therapy, induced significantly lower anti‐Spike antibody levels following the fourth vaccine dose compared to the other medication groups (median 5620 BAU/ml [IQR 2840–10,248] vs. 8910 BAU/ml [5417–13,266], *p* < 0.001). In the hybrid group, CD patients had a lower serologic response following Omicron infection than the other diagnoses (median 13,415 BAU/ml [IQR 8081–26,966] vs. 27,122 BAU/ml [12,361–40,962], *p* = 0.008).

Adverse events (AEs) were reported by 46% and 35% of patients after the third and fourth vaccine dose, respectively, and by 72% of healthy controls after the third dose. Patients and controls had a comparable safety profile, and no serious AEs were reported.

**Conclusion:** Repeated vaccination improve humoral immunity in IMID patients, supporting regular booster vaccination. Hybrid immunisation induces a humoral response higher than four SARS‐CoV‐2 vaccine doses. No difference in humoral immune responses was found between vaccine types, and no new safety signals emerged. Further studies are needed to address the clinical importance of hybrid immunity against the Omicron variants and how hybrid immunisation should be accommodated into an optimal regime for booster vaccinations in this vulnerable population.

**Reference**

1. Clinialtrials.gov NCT04798625.

**Disclosure:** KHB reports funding from Akershus University Hospital and speaker bureaus from Janssen‐Cilag. TTK reports consulting fees from AbbVie, Biogen, Celltrion, Eli Lilly, Gilead, Mylan, Novartis, Pfizer, Sandoz and Sanofi, and speakers bureaus from Amgen, Celltrion, Egis, Evapharma, Ewopharma, Hikma, Oktal, Sandoz and Sanofi. JJ reports grants from Boehringer‐Ingelheim, speakers bureaus from AbbVie/Abbott, Bristol‐Myers Squibb, Galapagos, Gilead, Janssen, Pfizer, Roche, Sandoz and Takeda, and participation on advisory board for AbbVie/Abbott, Bristol‐Myers Squibb, Galapagos, Gilead, Janssen, Pfizer, Roche, Sandoz and Takeda. LAM reports funding from KG Jebsen foundation, University of Oslo and Oslo University Hospital, grants from the Coalition of Epidemic Preparedness Innovations CEPI and speakers bureaus from Novartis and Cellgene. GG reports speaker bureaus from Bayer, Sanofi and ThermoFisher, and participation on advisory board from AstraZeneca. EAH reports speakers bureaus from UCB and consultant fees from AbbVie, Boehringer‐Ingelheim, Eli Lilly, Gilead. JTV reports grant from the Coalition of Epidemic Preparedness Innovations (CEPI). GLG reports speakers bureaus from AbbVie/Abbott, Galapagos, Pfizer, UCB, and participation on advisory board from AbbVie/Abbott, Galapagos, Pfizer and UCB. KKJ reports speakers bureaus from Bristol‐Myers Squibb and Roche. HSØ, ATT, TTT, JS, IJ, IEC, GBK, DJW, AC, SM, SAP, and SWS report no disclosure.

## LB14 | DUPILUMAB IMPROVES HISTOLOGICAL AND ENDOSCOPIC FEATURES OF EOSINOPHILIC OESOPHAGITIS IN CHILDREN AGED 1–11 YEARS IN THE PHASE 3 EOE KIDS TRIAL

M. Chehade ^1^, E. S. Dellon^2^, J. M. Spergel^3^, M. E. Rothenberg^4^, R. Liu^5^, L. P. Mannent^6^, E. Laws^7^, E. Mortensen^5^, J. Maloney^5^, E. McCann^5^, F. A. Khokar^5^, B. Beazley^5^, L. Glotfelty^7^, A. Shabbir^5^

^1^ *Mount Sinai Center for Eosinophilic Disorders, Icahn School of Medicine at Mount Sinai, New York, New York, USA*, ^2^ *University of North Carolina School of Medicine, Chapel Hill, North Carolina, USA*, ^3^ *Children's Hospital of Philadelphia, Philadelphia, Pennsylvania, USA*, ^4^ *Cincinnati Children's Hospital Medical Center and University of Cincinnati College of Medicine, Cincinnati, Ohio, USA*, ^5^ *Regeneron Pharmaceuticals, Inc., Tarrytown, New York, USA*, ^6^ *Sanofi, Chilly‐Mazarin, France*, ^7^ *Sanofi, Bridgewater, New Jersey, USA*

**Contact E‐Mail Address**: mirna.chehade@mssm.edu

**Introduction:** Eosinophilic oesophagitis (EoE) is a chronic, progressive, type 2 inflammatory disease of the oesophagus with no approved treatments for children under 12 years of age. Dupilumab, a fully human monoclonal antibody, blocks the shared receptor component for interleukin (IL)‐4/IL‐13, key and central drivers of type 2 inflammation. In phase 3 LIBERTY‐EoE‐TREET, dupilumab demonstrated clinically meaningful improvements in histological, symptomatic, and endoscopic outcomes and was well tolerated in adolescents and adults with EoE. The phase 3 EoE KIDS trial (NCT04394351) was designed to evaluate efficacy, safety, and tolerability of dupilumab versus placebo in paediatric patients aged 1–11 years, with active EoE unresponsive to proton pump inhibitors (PPI).

**Aims and Methods:** In Part A, 102 participants were randomized 1:1:1 to subcutaneous (SC) dupilumab higher‐ (*n* = 37), or lower‐exposure dose (*n* = 31), or matching placebo (*n* = 34) for 16 weeks. Key inclusion criteria include: documented diagnosis of EoE unresponsive to ≥8‐week PPI and baseline oesophageal intraepithelial eosinophil (eos) count of ≥15 eos/high‐power field (hpf) in ≥2 of three regions. Key exclusion criteria include body weight <5 or ≥60 kg at screening, eosinophilic gastroenteritis, and non‐EoE causes of oesophageal eosinophilia.

**Results:** At Week 16, 68% of children on higher‐dose and 58% on lower‐dose dupilumab achieved the primary endpoint of histological remission (peak oesophageal intraepithelial eosinophil count ≤6 eos/hpf) compared to 3% of children on placebo (both *P* < 0.0001). Children receiving higher‐dose dupilumab experienced the following at Week 16: 86% reduction in peak oesophageal intraepithelial eos count from baseline compared to 21% increase for placebo (*P* < 0.0001); 3.5‐point reduction in EoE Endoscopic Reference Score (EREFS) from baseline compared to 0.3‐point increase for placebo (*P* < 0.0001); numerical improvement compared to placebo in the proportion of days with ≥1 signs of EoE from baseline, as reported by caregivers (Paediatric EoE signs/symptoms questionnaire [PESQ‐C]); 3.09 percentile increase in body weight for age from baseline compared to 0.29 for placebo. Results were generally comparable in the lower‐dose group. In Part A, overall rates of adverse events (AEs) were 79% for dupilumab and 91% for placebo. AEs more commonly observed with dupilumab compared to placebo included COVID‐19, rash, and headache. AEs leading to study drug discontinuation occurred in 0% with dupilumab and 6% with placebo.

**Table**. Higher‐dose dupilumab versus placebo in paediatric patients with EoE

**Table:** 

	Placebo (*n* = 34)	Dupilumab higher‐dose (*n* = 37)
Proportion achieving peak oesophageal intraepithelial eos count ≤6 eos/hpf^a^	3%	68%
*p* value versus placebo		<0.0001
Percentage change from baseline in peak eos count^a^	+21%	−86%
*p* value versus placebo		<0.0001
Absolute change from baseline in EoE EREFS total score^b^	+0.3	−3.5
*p* value versus placebo		<0.0001
Percentile change from baseline in body weight for age	+0.29	+3.09

^a^ Punch biopsies from three oesophageal regions (proximal, mid, distal) at screening and Week 16 for histology.

^b^ Endoscopies at screening and Week 16, proximal and distal oesophageal regions scored for oedema, rings, exudates, furrows, strictures. The overall score ranges from 0 to 18; higher scores = greater severity.

**Conclusion:** This phase 3 trial assessing dupilumab versus placebo in children aged 1–11 years with active EoE met its primary endpoint of histological disease remission at 16 weeks with both higher‐ and lower‐dose weight‐tiered regimens. Analysis of secondary outcomes in the higher‐dose group demonstrated improvements with dupilumab in histological and endoscopic disease aspects. Safety results were generally consistent with the known safety profile of dupilumab.

**Disclosure:** **Chehade M**: Adare Pharma Solutions/Ellodi, Allakos, AstraZeneca, BMS, Regeneron Pharmaceuticals, Inc., Sanofi, Shire/Takeda, Phathom – consultant; Adare Pharma Solutions/Ellodi, Allakos, AstraZeneca, Danone, Regeneron Pharmaceuticals, Inc., Shire/Takeda – research funding. **Dellon ES**: Abbott, AbbVie, Adare Pharma Solutions, Aimmune Therapeutics, Allakos, Amgen, Arena Pharmaceuticals, AstraZeneca, Avir Pharma, Biorasi, BMS, Calypso Biotech, Celgene, Calyx, Celldex Therapeutics, Eli Lilly, Ellodi Pharmaceuticals, EsoCap, Gossamer Bio, GSK, Landos Biopharma, Morphic Therapeutic, Parexel, Receptos/BMS, Regeneron Pharmaceuticals, Inc., Robarts Clinical Trials, Salix Pharmaceuticals, Sanofi, Shire/Takeda – consultant; Adare Pharma Solutions, Allakos, AstraZeneca, BMS, Celgene, Ellodi Pharmaceuticals, GSK, Meritage, Miraca Life Sciences, Nutricia, Receptos/BMS, Regeneron Pharmaceuticals, Inc., Shire/Takada – research funding; Allakos, Banner Pharmaceuticals, Holoclara – educational grant. **Spergel JM**: Allakos, DBV Technologies, Novartis, Regeneron Pharmaceuticals, Inc., Shire/Takeda – consultant; DBV Technologies, Regeneron Pharmaceuticals, Inc. – grant support. **Rothenberg ME**: Allakos, AstraZeneca, BMS, ClostraBio, PulmOne, Spoon Guru ‒ consultant; ClostraBio, PulmOne, Spoon Guru ‒ equity interest; Teva Pharmaceuticals ‒ royalties from reslizumab; Mapi Research Trust ‒ royalties from PEESSv2; UpToDate ‒ royalties; inventor of patents owned by Cincinnati Children's Hospital. **Liu R, Mortenson E, Maloney J, McCann E, Khokhar FA, Beazley B, Shabbir A**: Regeneron Pharmaceuticals, Inc. – employees and shareholders. **Mannent LP, Laws E, Glotfelty L**: Sanofi – employees, may hold stock and/or stock options in the company.

## LB15 | COVID‐19 VACCINE‐INDUCED ANTIBODY AND T CELL RESPONSES IN IMMUNOSUPPRESSED PATIENTS WITH INFLAMMATORY BOWEL DISEASE AFTER THE THIRD VACCINE DOSE

J. Alexander ^1^, Z. Liu^1^, D. Munoz Sandoval^1^, C. Reynolds^1^, H. Ibraheim^1^, S. Anandabaskaran^2^, A. Saifuddin^2^, R. Castro Seoane^1^, N. Anand^1^, R. Nice^3^, C. Bewshea^3^, A. D'Mello^1^, L. Constable^1^, G. Jones^4^, S. Balarajah^1^, F. Fiorentino^5^, S. Sebastian^6^, P. Irving^7^, L. Hicks^1^, H. Williams^1^, A. Kent^8^, R. Linger^9^, M. Parkes^10^, K. Kok^11^, K. V. Patel^12^, J. Teare^1^, D. Altmann^1^, J. Goodhand^3^, A. L. Hart^2^, C. Lees^4^, R. Boyton^1^, N. Kennedy^3^, T. Ahmad^3^, N. Powell^1^

^1^ *Imperial College London, London, UK*, ^2^ *Gastroenterology, St Mark's Hospital and Academic Institute, Harrow, UK*, ^3^ *University of Exeter, Exeter, UK*, ^4^ *Edinburgh IBD Unit, University of Edinburgh, Edinburgh, UK*, ^5^ *King's College London, London, UK*, ^6^ *IBD Unit, Hull University Teaching Hospitals NHS Trust, Hull, UK*, ^7^ *Department of Gastroenterology, Guy's and St Thomas' Hospital, London, UK*, ^8^ *Gastroenterology, Kings College Hospital NHS Trust, London, UK*, ^9^ *NIHR Bioresource, University of Cambridge, Cambridge, UK*, ^10^ *Inflammatory Bowel Disease Research Group, Addenbrooke's Hospital, Cambridge, UK*, ^11^ *Bart's Health, London, UK*, ^12^ *Gastroenterology, St George's Hospital, London, UK*

**Contact E‐Mail Address**: jamesalexander@doctors.org.uk

**Introduction:** COVID‐19 vaccine‐induced antibody responses are reduced in patients with inflammatory bowel disease (IBD) taking infliximab or tofacitinib after two vaccine doses (1, 2). Breakthrough infection is more common in IBD patients receiving infliximab compared to vedolizumab after two vaccine doses (2). There are limited data on humoral and cell‐mediated anti‐SARS‐CoV‐2 immunity in patients with IBD compared to non‐immunosuppressed healthy controls after three COVID‐19 vaccine doses.

**Aims and Methods:** We sought to determine whether immunosuppressive treatments were associated with reduced antibody and T cell responses after a third COVID‐19 vaccine dose. 352 adults (72 healthy controls and 280 IBD) from the prospectively recruited VIP study cohort were sampled 28–49 days after a third dose of SARS‐CoV‐2 vaccine. IBD medications studied included thiopurines (*n* = 65), infliximab (*n* = 46), thiopurine/infliximab combination therapy (*n* = 49), ustekinumab (*n* = 44), vedolizumab (*n* = 50) or tofacitinib (*n* = 26). SARS‐CoV‐2 spike antibody binding and T cell responses were measured.

**Results:** Geometric mean [geometric SD] anti‐S1 RBD antibody concentrations increased in all study groups following a third dose of vaccine, but were significantly lower in patients treated with infliximab (2736.8 U/ml [4.3]; *p* < 0.0001), infliximab and thiopurine combination (1818.3 U/ml [6.7]; *p* < 0.0001) and tofacitinib (8071.5 U/ml [3.1]; *p* = 0.0018) compared to controls (16,774.2 U/ml [2.6]). There were no significant differences in anti‐S1 RBD antibody concentrations between control subjects and thiopurine (12,019.7 U/ml [2.2]; *P* = 0.099), ustekinumab (11,089.3 U/ml [2.8]; *P* = 0.060), nor vedolizumab treated patients (13,564.9 U/ml [2.4]; *p* = 0.27). In multivariable modelling, lower anti‐S1 RBD antibody concentrations were independently associated with infliximab (geometric mean ratio 0.15, 95% CI 0.11–0.21, *p* < 0.0001), tofacitinib (0.52, 95% CI 0.31–0.87, *p* = 0.012) and thiopurine (0.69, 95% CI 0.51–0.95, *p* = 0.021), but not with ustekinumab (0.64, 95% CI 0.39–1.06, *p* = 0.083), or vedolizumab (0.84, 95% CI 0.54–1.30, *p* = 0.43). Previous SARS‐CoV‐2 infection (1.58, 95% CI 1.22–2.05, *p* = 0.00056) and older age (0.88, 95% CI 0.80–0.97, *p* = 0.0073) were independently associated with higher and lower anti‐S1 antibody concentrations respectively. However, antigen specific T cell responses were similar in IBD patients in all treatment groups studied, except for recipients of tofacitinib without evidence of previous infection, where T cell responses were significantly reduced relative to healthy controls (*p* = 0.021).

**Conclusion:** A third dose of COVID‐19 vaccine induced a boost in antibody binding in immunosuppressed patients with IBD, but these responses were reduced in patients taking infliximab, infliximab/thiopurine combination and tofacitinib therapy. Tofacitinib was also associated with reduced T cell responses. These findings support continued prioritisation of immunosuppressed groups for further booster dosing, particularly those on Janus Kinase (JAK) inhibitors who have attenuation of both serological and cell‐mediated vaccine‐induced immunity.

Financial support was provided as a Research Grant by Pfizer Ltd.

**References**

1. Alexander JL, Kennedy NA, Ibraheim H, Anandabaskaran S, Saifuddin A, Seoane RC, et al. COVID‐19 vaccine‐induced antibody responses in immunosuppressed patients with inflammatory bowel disease (VIP): a multicentre, prospective, case‐control study. *Lancet Gastroenterol Hepatol*. 2022;7:342–52.
2. Lin S, Kennedy NA, Saifuddin A, Sandoval DM, Reynolds CJ, Seoane RC, et al. Antibody decay, T cell immunity and breakthrough infections following two SARS‐CoV‐2 vaccine doses in inflammatory bowel disease patients treated with infliximab and vedolizumab. *Nat Commun*. 2022;13(1):1379.

**Disclosure:** NK reports grants from AbbVie, Biogen, Celgene, Celtrion, Galapagos, MSD, Napp, Pfizer, Pharmacosmos, Roche and Takeda, consulting fees from Amgen, Bristol‐Myers Squibb, Falk, Janssen, Mylan, Pharmacosmos, Galapagos, Takeda and Tillotts, personal fees from Allergan, Celltrion, Falk, Ferring, Janssen, Pharmacosmos, Takeda, Tilllotts, Galapagos, and support for attending meetings from AbbVie, Falk and Janssen. AS has received travel expense support from Janssen. SS reports grants from Takeda, Abbvie, Tillots Pharma, Janssen, Pfizer, Biogen and personal fees from Takeda, Abbvie, Janssen, Pharmacocosmos, Biogen, Pfizer, Tillots Pharma and Falk Pharma. AH reports payment or honoraria for lectures, presentations, speakers bureaus, manuscript writing or educational events from AbbVie, AZ, Atlantic, Bristol‐Myers Squibb, Celltrion, Falk, Galapogos, Janssen, MSD, Napp Pharmaceuticals, Pfizer, Pharmacosmos, Shire and Takeda and Global Steering Committee for Genentech, support for attending meetings from Abbvie, Takeda and Janssen, and Participation on a Data Safety Monitoring Board or Advisory Board for AbbVie, AZ, Atlantic, Bristol‐Myers Squibb, Galapogos, Janssen, Pfizer and Takeda. PI reports grants and personal fees from Celltrion, Takeda, Pfizer, Galapagos, grants from MSD, personal fees from Gilead, Abbvie, Janssen, BMS, Lilly, and Arena. MP receives unrestricted educational grants from Pfizer for genetic analyses to support the IBD BioResource and speaker fees from Janssen. GJ has received grants from Wellcome Trust and ECCO, speaker fees from Takeda, Ferring and Janssen and support for attending meetings and/or travel from Ferring. KK reports payment or honoraria for lectures, presentations, speakers bureaus, manuscript writing or educational events from Janssen and Ferring, support for attending meetings and/or travel Janssen and Takeda and Participation on a Data Safety Monitoring Board or Advisory Board from Janssen and Predict Immune. KP reports payment or honoraria for lectures, presentations, speakers bureaus, manuscript writing or educational events from Abbvie, DrFalk, Janssen, PreddictImmune, Takeda, support for attending meetings and/or travel from Abbvie, Ferring, Janssen, Tillots and Participation on a Data Safety Monitoring Board or Advisory Board from Abbvie, Galapagos and Janssen. AK reports consulting fees from Janssen, payment or honoraria for lectures, presentations, speakers bureaus, manuscript writing or educational events from Pfizer and Takeda, support for attending meetings and/or travel from Janssen, Tillots and Norgine, Participation on a Data Safety Monitoring Board or Advisory Board from Abbvie. LH reports support for attending meetings and/or travel from Abbvie. CL reports a Future Leaders Fellow award from UKRI, personal consulting fees from Galapagos, Abbvie, Takeda, Pfizer, Janssen, Iterative Scopes and institutional consulting fees from Trellus Health, personal fees from Galapagos, Abbvie, Takeda, Pfizer, Janssen, GSK, Gilead, Fresnius Kabi, Ferring and Dr Falk, and support for attending meetings from Galapagos, Abbvie, Takeda, Pfizer, Janssen, GSK, Gilead, Fresnius Kabi, Ferring and Dr Falk. RB and DA are members of the Global T cell Expert Consortium and have consulted for Oxford Immunotec. JG reports grants from F. Hoffmann‐La Roche AG, grants from Biogen Inc, grants from Celltrion Healthcare, grants from Galapagos NV, non‐financial support from Immundiagnostik, during the conduct of the study. TA reports grant funding from Pfizer to his institution to deliver this study, grants from Celltrion, Roche, Takeda, Biogen and Galapagos and honoraria for lectures from Takeda and Roche. Financial support for the VIP study was provided as a Research Grant by Pfizer Ltd and NP is the PI on this grant andl has received research grant(s) from Bristol Myers Squibb. NP reports personal fees from Takeda, Janssen, Pfizer, Bristol‐Myers Squibb, Abbvie, Roche, Lilly, Allergan, and Celgene, and has served as a speaker/advisory board member for Abbvie, Allergan, Bristol Myers Squibb, Celgene, Falk, Ferring, Janssen, Pfizer, Tillotts, Takeda and Vifor Pharma.

